# Microfluidic Airborne Metal Particle Sensor Using Oil Microcirculation for Real-Time and Continuous Monitoring of Metal Particle Emission

**DOI:** 10.3390/mi12070825

**Published:** 2021-07-14

**Authors:** Jong-Seo Yoon, Jiwon Park, Hye-Rin Ahn, Seong-Jae Yoo, Yong-Jun Kim

**Affiliations:** School of Mechanical Engineering, Yonsei University, 50 Yonsei-ro, Seodaemun-gu, Seoul 03722, Korea; jsyoon129@yonsei.ac.kr (J.-S.Y.); prion1548@yonsei.ac.kr (J.P.); hyerinahn@yonsei.ac.kr (H.-R.A.); sj92@yonsei.ac.kr (S.-J.Y.)

**Keywords:** microfluidics, microcirculation, particle-to-liquid collection, airborne metal particle, continuous and real-time monitoring, capacitive detection

## Abstract

Airborne metal particles (MPs; particle size > 10 μm) in workplaces result in a loss in production yield if not detected in time. The demand for compact and cost-efficient MP sensors to monitor airborne MP generation is increasing. However, contemporary instruments and laboratory-grade sensors exhibit certain limitations in real-time and on-site monitoring of airborne MPs. This paper presents a microfluidic MP detection chip to address these limitations. By combining the proposed system with microcirculation-based particle-to-liquid collection and a capacitive sensing method, the continuous detection of airborne MPs can be achieved. A few microfabrication processes were realized, resulting in a compact system, which can be easily replaced after contamination with a low-priced microfluidic chip. In our experiments, the frequency-dependent capacitive changes were characterized using MP (aluminum) samples (sizes ranging from 10 μm to 40 μm). Performance evaluation of the proposed system under test-bed conditions indicated that it is capable of real-time and continuous monitoring of airborne MPs (minimum size 10 μm) under an optimal frequency, with superior sensitivity and responsivity. Therefore, the proposed system can be used as an on-site MP sensor for unexpected airborne MP generation in precise manufacturing facilities where metal sources are used.

## 1. Introduction

The development of automotive technologies has been rapidly expanding owing to an increased focus on reducing environmental impact [1,2]. Various electronics have been embedded into autonomous systems for different operations, such as supplying power and controlling steering systems [3]. Following this ongoing trend regarding driving devices, there is a demand for the miniaturization and integration of electronics to achieve spatial savings, low power consumption, and high electrical performance [4,5]. For packaged semiconductor chips, rechargeable batteries, and other components that aim at the same goal, greater safety and quality are essential [6]. However, tremendous effort should be invested because these electronic components have a wide range of production failures [7]. Among the common issues, airborne metal particles (MPs; particles larger than 10 μm) at manufacturing sites are one of the root causes of the degradation of the lifespan and reliability of the products, because they induce internal short circuits or stress points [8,9]. There have been advances in manufacturing procedures such as encapsulation packaging, electrode welding, and cutting; however, micrometer-sized MPs break down from the metal sources, scatter in the air, and settle on multiple spots that may be electrically connected [10,11]. In addition, when the deterioration of manufacturing machinery begins, the numbers and sizes of MPs become larger [12,13]. Thus, airborne MPs must be monitored to improve the current production yield.

Currently, compositional analysis instruments have been widely adopted to determine the level of contamination and particle composition using an X-ray and inductively coupled plasma techniques [14,15,16]. The samples used for analysis must be collected in advance and pre-treated. Adhesive films are placed at points of potential emitters in manufacturing lines and are routinely replaced [17]. Manufacturers can obtain quantitative (i.e., number and size) and qualitative (i.e., element map) information through sample analysis [18]. Although the current analytical system can determine the approximate contamination without temporal resolution, the procedures are time-consuming and complex. Moreover, these instruments are expensive, have a large volumetric size, and require regular maintenance, which leads to limitations in monitoring the MPs and the points of interest on a real-time basis. Thus, inexpensive compact sensors are required for on-site monitoring and to establish a dense monitoring network throughout the worksites.

Instead of monitoring MPs in the air, many studies have been performed to detect MPs in a hydraulic oil [12]. These attempts have been based on electrical detection, such as inductive or capacitive techniques [19,20,21,22,23,24,25,26,27,28]. Most contemporary laboratory-grade sensors have successfully detected MPs in sizes ranging from meso- to micro-scale in real-time. Among them, a sensor based on a capacitive detection technique was developed based on a microfluidic platform with a greater sensitivity than the inductive method [28]. They demonstrated real-time monitoring of MPs up to 10 µm with a micro-sized sensing volume. However, previous studies have only focused on detecting MPs in hydraulic oil without a particle collection system. To monitor airborne MPs, though they are not easily detected in oil, a microfluidic electrical detection system with a particle collection system should be devised.

However, it is difficult to directly measure airborne MPs owing to their heavy gravity. Moreover, cumulative particle detection, which identifies the increasing steps of signals depending on particle accumulation, is not an approved method for monitoring MPs [29,30]. This detection mechanism can cause short circuits on electrical detection elements and hence, require periodic cleaning. To pass the particles over the detection element, a viscous liquid as a particle carrier fluid is essential. Hence, because a sensing element is in the liquid phase, a particle-to-liquid collection method must be incorporated. Several approaches have been proposed, and many studies in the field of bioaerosol analysis have shown continuous monitoring based on this collection method [31,32]. Despite the considerable progress, they are currently not applicable for monitoring airborne MPs because they are bulky and complicated. This is because their liquid supply and exhaust system, which exist independently, occupy a lot of space to maintain a liquid surface for particle inflow. Therefore, an integrated microfluidic system with circulation-based particle delivery from the air to the liquid and electrical detection is required for the continuous and sensitive monitoring of MPs.

Herein, we present the integration of a microfluidic channel and a capacitive particle detection element into a single chip that can detect airborne MPs continuously in real-time. The proposed system is composed of a microcirculation-based particle delivery system and a particle detection element. Airborne MPs settle in the liquid and are delivered to the detection region by microcirculation. A coplanar capacitive sensor used for particle detection was adopted owing to its high sensitivity and simple fabrication with a microchannel. Microfabricated interdigitated electrodes (IDEs) with a coplanar configuration were realized at the bottom of the relatively small points in the microchannel. The capacitive change generated by individual particles greatly depends on the dielectric properties, particle size, particle *z*-axis position from the electrodes, and the applied AC voltage. By adopting hydraulic oil as the particle carrier fluid, we obtained the advantages of particle detection and delivery. Furthermore, the oil reservoir plays the role of a tank for circulation; however, it is also a place where the detected particles are sunk or accumulated. If the MP detection chip is highly contaminated by particles after operating for a long time, it can be disposed and replaced. Our chip is low in cost because it is fabricated in a batch process and easily assembled with other microelectromechanical system (MEMS)-compatible parts. Thus, we demonstrate the feasibility of monitoring airborne MPs by integrating these novel functions. Our system is expected to be widely applicable in workplaces that must deal with metal sources, because it facilitates immediate action and determines the causes of defect points.

## 2. Principle of Particle Detection

**Figure 1:**
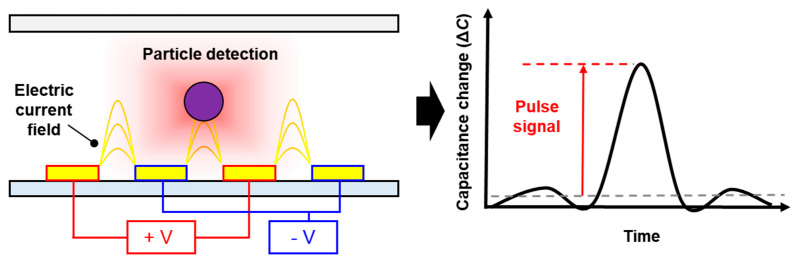
Simplified schematic diagram for particle detection under applied AC voltage.

The capacitive sensing technique on a microfluidic platform to measure the variations generated by particles is mainly based on the contrast of dielectric properties (e.g., permittivity and conductivity). By placing the electrodes close to the particle matter trajectory, the particles passing through the detection region are inevitably affected by an AC electrical field. Based on this phenomenon, such variations are directly related to the interaction of the created electric field as well as the occupied volume fraction between the matter and medium in the detection region. Therefore, in this study, the presence of MPs is displaced by the equivalent capacitance and measured as a positive pulse, as illustrated in Figure 1. Our particle detection element was performed using an LCR meter, which is a high-precision instrument, rather than a lock-in amplifier, owing to its interface convenience. The capacitance variations can be equivalently calculated by measuring the impedance of the capacitor.

Impedance (Z) is defined as the ratio of two electrical characteristics, one of which depends on variations in the dielectric properties (permittivity and conductivity) of the system (i.e., electric current response (I)), and the other is the frequency-dependent excitation signal with a small voltage (AC voltage (V)) [33].

(1) $$Z\left(j\omega \right)=\frac{V\left(j\omega \right)}{I\left(j\omega \right)}{=Z}_{\mathrm{re}}{+\mathrm{jZ}}_{\mathrm{im}}$$

where $j^{2}=-1$, ω is the angular frequency; $Z_{\mathrm{re}}$ is the real part (resistance), and $Z_{\mathrm{im}}$ is the imaginary part (conductance) of the complex impedance.

Based on the above equation, according to the principle of the specific mode in the LCR meter used in this study, the impedance is calculated as follows [34]:

(2) $$Z=\frac{{\mathrm{RX}}_{C}{}^{2}}{R^{2}{+X}_{C}{}^{2\;}}+j\frac{R^{2}X_{C}}{R^{2}{+X}_{C}{}^{2}}$$

where R is the resistance, and $X_{C}$ is the capacitive reactance. $X_{C}$ is given as follows:

(3) $$X_{C}=\frac{1}{{j\omega C}_{\mathrm{eq}}}$$

Therefore, the equivalent capacitance ($C_{\mathrm{eq}})$ can be derived as follows:

(4) $$C_{\mathrm{eq}}=\frac{1}{\omega }\cdot \frac{Z_{\mathrm{im}}}{{\left(Z_{\mathrm{re}}\right)}^{2}+{\left(Z_{\mathrm{im}}\right)}^{2}}$$

**Figure 2:**
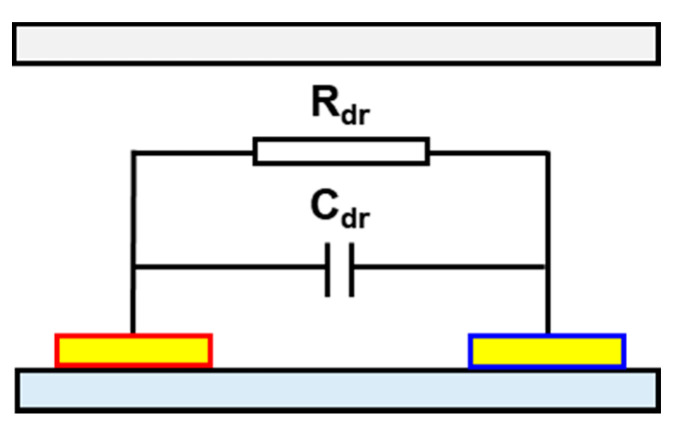
Equivalent electrical circuit of the detection region.

A simplified electrical equivalent circuit model is presented in Figure 2 to illustrate the electrical variations in the detection region. The measured impedance ($Z_{\mathrm{total}})$ can be expressed as a part of capacitance (C) and resistance (R) in parallel. It includes the capacitance ($C_{\mathrm{dr}})$ and resistance ($R_{\mathrm{dr}})$ around the electrodes in the detection region. The measured impedance is expressed as follows:

(5) $$Z_{\mathrm{total}}{=R}_{\mathrm{dr}}+\frac{1}{{j\omega C}_{\mathrm{dr}}}$$

In practice, the dielectric behavior varies depending on the operating conditions. The permittivity is given as follows:

(6) $$\tilde{\varepsilon }=\varepsilon -j\frac{\sigma }{\omega }$$

where $\tilde{\varepsilon }$ is the complex permittivity, and σ is the conductivity.

Therefore, the impedance ($Z_{0})$ of the capacitor in the empty site, which is filled with the medium, is given as follows:

(7) $$Z_{0}=\frac{1}{j\omega {\tilde{\varepsilon }}_{\mathrm{med}}G_{f}}$$

where ${\tilde{\varepsilon }}_{\mathrm{med}}$ is the complex permittivity of the medium, and $G_{f}$ is the geometric constant based on the configuration of the electrodes. In this study, we adopted an interdigital shape for the electrode configuration. 

In the case of a particle in the medium, the equivalent complex permittivity (${\tilde{\varepsilon }}_{\mathrm{mix}}$) is well described using the Maxwell mixture theory because of the interaction between the particles and the medium [35].

(8) $${\tilde{\varepsilon }}_{\mathrm{mix}}={\tilde{\varepsilon }}_{\mathrm{med}}\frac{1+2{\Phi \tilde{f}}_{\mathrm{CM}}}{1-{\Phi \tilde{f}}_{\mathrm{CM}}}$$

where Φ is the volume fraction, and ${\tilde{f}}_{\mathrm{CM}}$ is the Clausius–Mossotti factor. Φ is the volume fraction of the occupied volume of the particles ($V_{\mathrm{ptc}}$) and the detection region ($V_{\mathrm{dr}})$ and is defined as

(9) $$\Phi =\frac{V_{\mathrm{ptc}}}{V_{\mathrm{dr}}}$$

and ${\tilde{f}}_{\mathrm{CM}}$ is the Clausius–Mossotti formula of the dielectric function defined as follows:

(10) $${\tilde{f}}_{\mathrm{CM}}=\frac{{\tilde{\varepsilon }}_{\mathrm{ptc}}-2{\tilde{\varepsilon }}_{\mathrm{med}}}{{\tilde{\varepsilon }}_{\mathrm{ptc}}+2{\tilde{\varepsilon }}_{\mathrm{med}}}$$

From (8)–(10), ${\tilde{\varepsilon }}_{\mathrm{mix}}$ is derived as follows:

(11) $${\tilde{\varepsilon }}_{\mathrm{mix}}={\tilde{\varepsilon }}_{\mathrm{med}}\frac{V_{\mathrm{dr}}({\tilde{\varepsilon }}_{\mathrm{ptc}}+2{\tilde{\varepsilon }}_{\mathrm{med}})+V_{\mathrm{ptc}}({\tilde{\varepsilon }}_{\mathrm{ptc}}-{\tilde{\varepsilon }}_{\mathrm{med}})}{V_{\mathrm{dr}}({\tilde{\varepsilon }}_{\mathrm{ptc}}+2{\tilde{\varepsilon }}_{\mathrm{med}})-V_{\mathrm{ptc}}({\tilde{\varepsilon }}_{\mathrm{ptc}}-{\tilde{\varepsilon }}_{\mathrm{med}})}$$

Therefore, the impedance ($Z_{\mathrm{mix}})$ of the capacitor in the medium containing the particle is given as follows:

(12) $$Z_{\mathrm{mix}}=\frac{1}{j\omega {\tilde{\varepsilon }}_{\mathrm{mix}}G_{f}}$$

Combining the above equations, the equivalent capacitance variation $\left(\Delta C_{\mathrm{eq}}\right)$ can be obtained as follows:

(13) $$\Delta C_{\mathrm{eq}}{=C}_{\mathrm{eq},\mathrm{mix}}-C_{\mathrm{eq},\mathrm{med}\;}=\frac{1}{\omega }\cdot \left\{\frac{Z_{\mathrm{mix},\mathrm{im}}}{{\left(Z_{\mathrm{mix},\mathrm{re}}\right)}^{2}+{\left(Z_{\mathrm{mix},\mathrm{im}}\right)}^{2}}-\frac{Z_{\mathrm{med},\mathrm{im}}}{{\left(Z_{\mathrm{med},\mathrm{re}}\right)}^{2}+{\left(Z_{\mathrm{med},\mathrm{im}}\right)}^{2}}\right\}$$

The amplitude of the capacitive pulse signal ($\Delta C_{\mathrm{eq}})$ depends on the excitation frequency (ω), occupied volume of the particles ($V_{\mathrm{ptc}}$), and mixture complex permittivity (${\tilde{\varepsilon }}_{\mathrm{mix}}$). Based on Equation (9), the sensing volume is crucial, along with the particle size to be detected. This is because the signal ($\Delta C_{\mathrm{eq}})$ can be increased accordingly if the particles occupy a larger volumetric proportion. Based on Equation (13), although increasing the frequency lowers the capacitance change and the noise floor, it is effective for distinguishing the frequency-dependent capacitance change in metals [36]. This is because the particles may be measured similarly to the baseline level of noise at low frequencies. Therefore, it is essential to optimize the operating conditions and geometrical factors of the structure (microfluidic channel and IDEs) to enhance the sensing performance.

## 3. Sensor Design and Fabrication

### 3.1. Operating Principle

**Figure 3:**
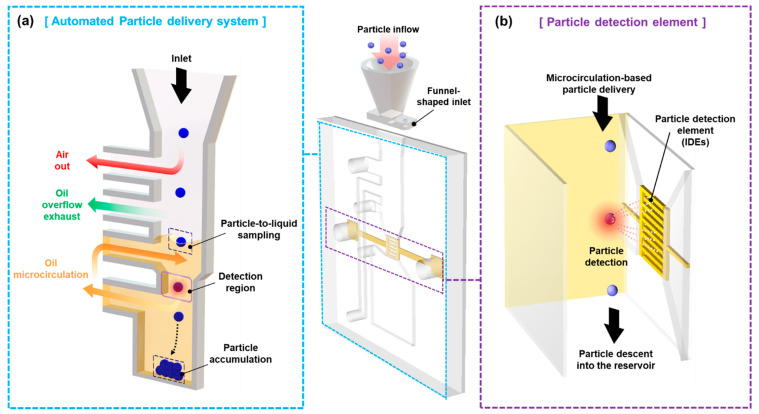
Schematic illustration of the proposed system. Our system consists of two sections: (**a**) an automated particle delivery system and (**b**) a particle detection element.

Our microfluidic sensor has two components: (a) an automated particle delivery system and (b) a particle detection element as shown in Figure 3. Airborne MPs are settled down to the funnel-shaped structures because of gravity. The particles drawn into the microchannel because of gravity or air outflow as vacuum air. The particles are impacted into the liquid and lose their velocity owing to the viscosity of the working fluid. Herein, the exhaust path exists to prevent oil overflow due to problems with particle clogging and pump pulsation. They are delivered to the particle detection region by microcirculation. For the detection region, the microfabricated IDEs used as the particle detection element perform the electrical excitation of the entire sensing volume and measure the electrical response in real-time. Subsequently, the particles pass through the detection region sink by escaping the mainstream of the microcirculation owing to their inertia and accumulating at the bottom of the oil reservoir.

### 3.2. Design of Automated Particle Delivery System 

A funnel-shaped structure was assembled in front of our microfluidic chip to induce airborne MPs toward the small opening of our microfluidic chip. This part helps in collecting scattered and free-falling particles with a wide geometrical design. The microfluidic chip connected to this structure was realized to perform two main roles for automated and continuous particle delivery: (i) collecting the particles that enter from the air to the viscous liquid by maintaining the liquid surface on the chip and (ii) delivering these particles to the particle detection element by internal microcirculation.

Hydraulic oil was adopted as the particle carrier liquid because it has a sufficient density (0.8 × $10^{3}$ kg/m^3^) to deliver the MPs (e.g., aluminum is 2.9 × $10^{3}$ kg/m^3^) and has a low-conductive media (the relative permittivity of the oil $\varepsilon_{o}\;\mathrm{is}\;2.6$) for visible dielectric contrast against the metals. The use of oil in a microchannel has already been validated as MPs are transported adequately to their detection elements [12]. Here, the MPs in chip operation are mainly influenced by the wall-induced lift force, mainstream drag force, and gravitational force [37,38]. Among them, the drag force and gravity owing to the dynamic viscosity of the oil and heavy gravity of the metal, respectively, have a dominant influence on the particle motion. Thus, our microfluidic device was placed at an angle to the ground to match the vectors of gravity and the mainstream toward the detection region. Through this placement, we could avoid unnecessary possibilities that could lead to chip failures in microfluidics, such as particle clogging or bottlenecks, and induce the MPs to go down to the oil reservoir section [39].

**Figure 4:**
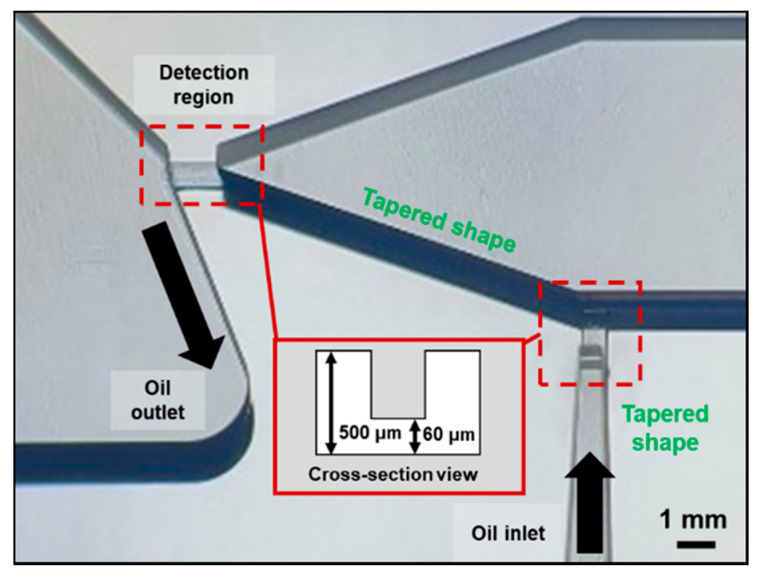
Optical photograph of the relatively small volumetric points (detection region and oil inlet) on the SU-8 mold.

For stable microcirculation of the viscous liquid (oil), we designed the overall structure of the microchannel to be rectangular in shape with a low aspect ratio (6000 μm × 500 μm) [40]. Based on the geometrical structure with margins, it allows precise flow control and enhances the stable operation. As an exception, only the dimensions of the required points (detection region and inlet of the oil microcirculatory system) were determined to be relatively low (2000 μm × 60 μm × 400 μm), as illustrated in Figure 4. Of the two points, the detection region was made to allow the particles to pass through closely to the particle detection element, and it occupied a large fraction of the sensing volume [41]. The other was matched with the dimensions of the detection region to minimize excessive mismatches in the flow rate of the microcirculation between the oil inlet and outlet. For each entrance line, the structure was designed in a tapered shape to avoid bottlenecks. It induces the incoming particles to the detection region to accelerate and pass through quickly. Because this microchannel is MEMS-compatible and easily fabricated, synergies can be obtained depending on the architecture of the microfabricated electrodes.

### 3.3. Design of Particle Detection Element

#### 3.3.1. Planar Electrode Array

The electrode configuration for creating an electric field has been mainly adopted as parallel or planar in particle detection sensor applications. Because electric current lines interact with the presence of matter in the media, a denser electric field is required. In the case of parallel electrodes, the distance between the electrodes is a key parameter because of the homogenous electric field [42]. Although a smaller gap could enhance the sensing performance, its geometrical dimension depends on the largest size of the particles to avoid such problems as particle clogging. Moreover, the fabrication procedures are complex because the electrodes are mechanically integrated at microscale intervals. Conversely, the other geometry is relatively simple because it can be fabricated on the same plane. In addition, the planar type exhibits higher sensing synergies with a microfluidic channel [43]. Owing to its non-uniform electric field, the peak signal becomes larger as the particles move closer toward the electrodes. In other words, the planar model could have the advantage of being able to detect small-sized particles with a more concentrated high-intensity electric field than the parallel one. Thus, the planar electrode array is a suitable configuration for our microfluidic platform.

#### 3.3.2. Interdigitated Microelectrodes

**Figure 5:**
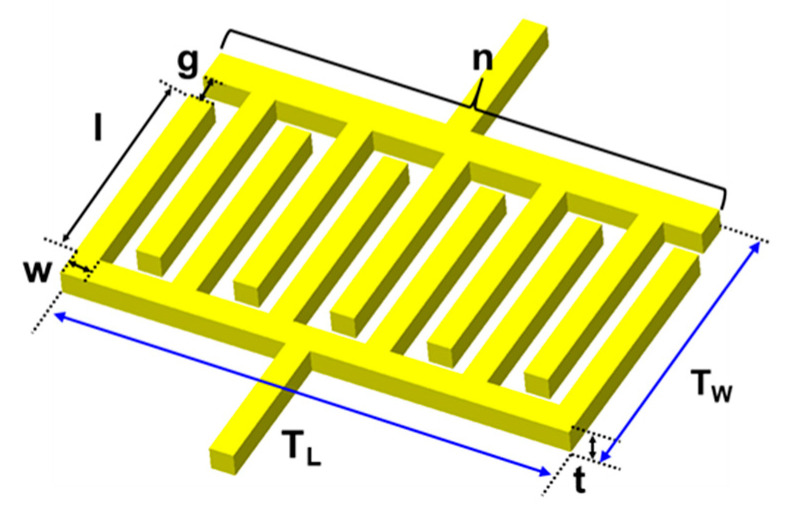
Simplified view of interdigitated electrodes with defined design factors.

Various studies have attempted to determine the optimal dimensions of electrodes using finite-element simulations validating that the shorter the distance between the electrodes, the larger the electric variations produced by the particles [30,44,45,46]. This is because the electric field line creates a strong boundary between the electrodes. Although the effect of geometrical optimization tends to be saturated, the order of the measured values changes considerably in terms of performance enhancement. For our microfluidic platform, the geometric design factors of planar electrodes need to be determined with data sampling frequency and particle residence time to detect the particles passing through the detection region in a short time. The design values of the required sensing area can be theoretically derived based on the determined flow rate of the working fluid and the geometric structure of the microchannel. Here, simply extending the distance between the electrodes would lower the intensity of the electric field. A single pair of coplanar electrodes could not sufficiently detect the particle behavior without increasing the applied voltage. Therefore, as illustrated in Figure 5, we adopted a shape for the electrodes, namely, several interdigitated pairs. The finger gap (g) and width (w) of the IDEs were 20 and 40 µm, respectively. They are the minimum linewidth and gap that can be achieved with the conventional lithography process using the film mask we have used. The finger length (l) was 390 µm. The width of the detection region and the finger length was matched so that the particle was affected by the electric field as soon as the particle enters from the entrance of the detection region. The number of fingers (n) was 13, chosen to ensure a sufficient sensing area. To detect at least two points or more with the sampling rate while the particle passes through the sensing volume, the sensing area was expanded with several pairs of fingers. The thickness of the electrodes were determined to be 0.21 µm to prevent unexpected bonding issues owing to the high flush between the microelectrodes and the microchannel [47]. Accordingly, the design values of the total electrode length ($T_{L}$) and width ($T_{w}$) were 1480 µm and 490 µm, respectively. The design factors of the IDE are listed in Table 1.

### 3.4. Sensor Fabrication

**Figure 6:**
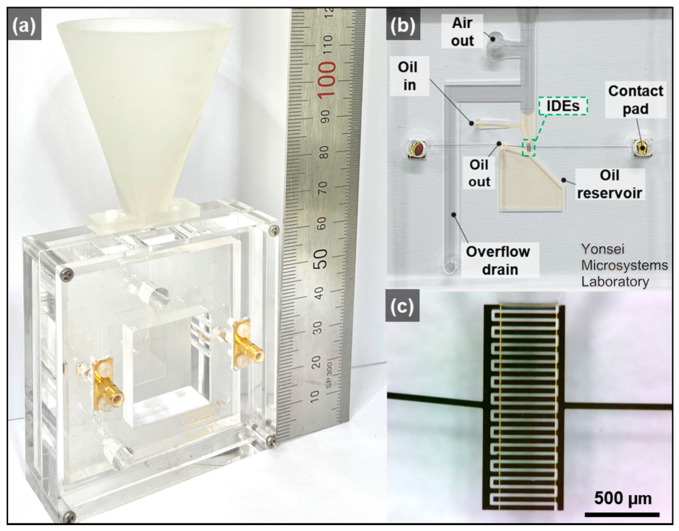
Optical paragraphs of (**a**) the proposed packaged system, (**b**) the microfluidic MP detection chip, and (**c**) the microfabricated interdigitated electrodes as a particle detection element.

Figure 6a presents photographs of the proposed packaged system. The prototype is compact (64 mm × 120 mm × 12 mm) for the on-site monitoring of airborne MPs at the points of interest. Using a semiconductor fabrication process, the microfluidic MP detection chip was realized on a single chip (52.5 mm × 52.5 mm × 7 mm), as presented in Figure 6b. The IDEs as particle detection elements could be identified and were placed in the detection region where the particles passed through, as presented in Figure 6c.

**Figure 7:**
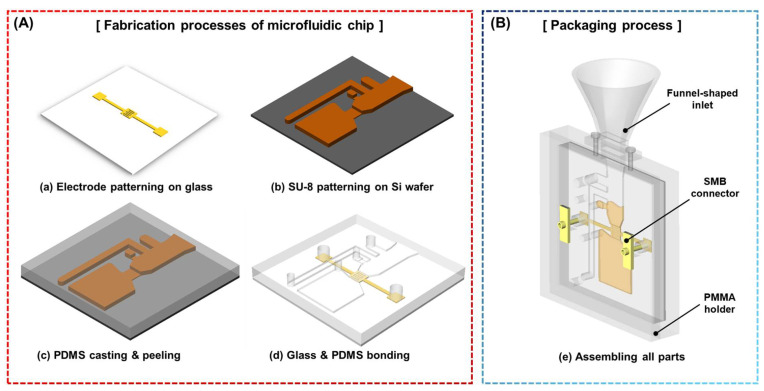
Simplified fabrication process of the proposed system, (**A**) the microfluidic MP detection chip, and (**B**) packaging.

Figure 7 illustrates the fabrication processes, which is explained in two schematic diagrams: (i) the microfluidic MP detection chip and (ii) the packaging process. IDEs were fabricated using photolithography. First, we deposited a thin metal layer (40/170 nm of Cr/Au) on a glass slide using an electron-beam evaporator. Subsequently, we spin-coated the positive-tone photoresist (AZ GXR 601, AZ Electronic Materials Co., Ltd., Wilmington, DE, USA) for 30 s at 3000 rpm and baked it softly on a hot plate for 1 min at 85 °C. After the glass was cooled to 25 °C, it was exposed to UV light with a printed film mask and was developed with a photoresist developer. To pattern the IDEs, wet etching was performed, following each etchant of the deposition layer in sequence (Figure 7a). In the case of fabricating the microchannel, the SU-8 negative tone photoresist, which has been extensively used in microfluidics, was adopted with a difference in height (500/60 µm). The first layer was coated (60 μm, model SU 8-50, Microchem Co., Westborough, MA, USA) on a Si wafer for 30 s at 1800 rpm and heated gently at two different temperatures (65 °C for 6 min and 95 °C for 20 min). Thereafter, it was exposed to UV light to define a relatively small aperture for the detection region and oil inlet. Because it was difficult to define a 500 µm layer at once, we proceeded with the deposition twice. The 250 µm layer was coated for 30 s at 1000 rpm, and a soft bake was conducted (65 °C for 30 min and 95 °C for 90 min). Next, the last 250 µm layer was formed in the same way as the first layer. After exposure to UV light, the baking process was conducted at 65 °C for 1 min and 95 °C for 40 min. Both exposed layers were developed using an SU-8 developer (Figure 7b). To realize the microchannel, Sylgard 184 (Dow Silicones Co., Midland, MI, USA) was mixed in advance at a standard cross-linker ratio of 10:1. Polydimethylsiloxane (PDMS) was cast to a thickness of 6.5 mm and placed in an environmental chamber for thermal aging (60 °C for 6 h). The PDMS was then peeled and punched using a biopsy (Figure 7c). To integrate the microchannel with a gold-patterned glass, the microchannel was exposed to oxygen plasma. Subsequently, an irreversible seal was formed by contacting the oxidized microchannel with an accurate alignment to the glass surface (Figure 7d).

In the case of packaging process (Figure 7e), the integrated microfluidic device was assembled using a polymethylmethacrylate (PMMA) holder. The rubber pad was laid for electrical insulation under the microchip covered with a case. The funnel-shaped inlet connected to the side was fabricated using a 3D printer in a single printout. SMB connectors for electrical connections, such as excitation and signal processing, were mounted on the case to fit the electrode pads.

## 4. Experiments

### 4.1. Preliminary Validation Test of Particle Detection Element with Optical Synchronization

The particle detection element is a crucial component for monitoring the delivered particles in microcirculation. Preliminary validation tests were implemented to characterize the performance of the element. In this experiment, a microfluidic rectangular sample chip (52.5 mm × 52.5 mm × 7 mm) that was embedded was used. The dimensions of the microchannel were identical to those of the detection region of the integrated system. 

The particle samples used in the experiment were aluminum particles (diameters of 10, 20, and 40 µm, RND Korea, Gwangmyeong, Korea). There were classified by size by using a sieve (10, 20, and 40 µm Pluristrainer, Pluriselect, Leipzig, Germany), and 3 mg was extracted using a microbalance (Precision balances WTC, Radwag, Radom, Poland). They were placed in a 50 mL centrifuge tube filled with hydraulic oil. The particle solutions were stirred for 5 min using a mixer (Maxshake-VM30, Daihan Scientific, Wonju, Korea). They were later drawn using a 5 mL syringe (Syringe Luer tip, Norm-Jet, Tuttlinger, Germany).

The test chip was connected to an LCR meter (E4980A, Agilent Technologies, Santa Clara, CA, USA) and the capacitance response was monitored using data processing software (LabVIEW, National Instruments, Austin, TX, USA), with the excitation voltage ($V_{\mathrm{ac}})$ and data sampling rate set to 15 V and 20 ms, respectively. To demonstrate that the signals were directly generated by particle inflow simultaneously, the detection region was observed using an optical microscope (UM-12, Vitiny, Kaohsiung, Taiwan). The particle solution was loaded using a syringe pump (KD-100, KD Scientific, Seoul, Korea) at a flow rate of 10 μL·min^−1^.

**Figure 8:**
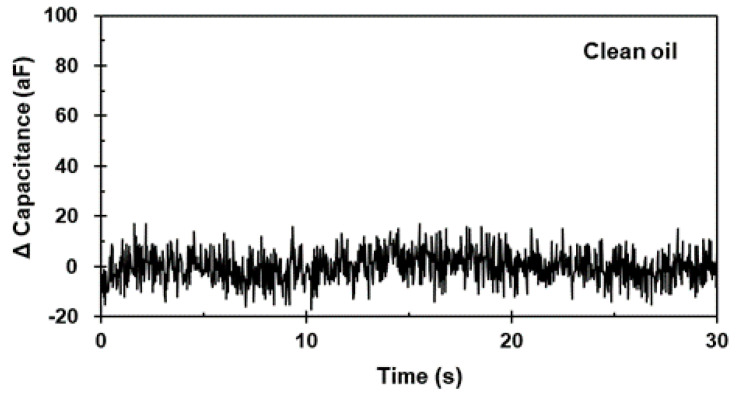
Measured capacitance signals for clean oil conditions.

Firstly, the test was performed in clean oil conditions without particle particles to ensure a noise floor level. Figure 8 presents the capacitance signal results for the clean oil conditions. Outlier signals, such as a peak signal, were not detected. The result shows a stable noise floor level, and a peak signal is not generated where the particle did not exist in the detection region.

**Figure 9:**
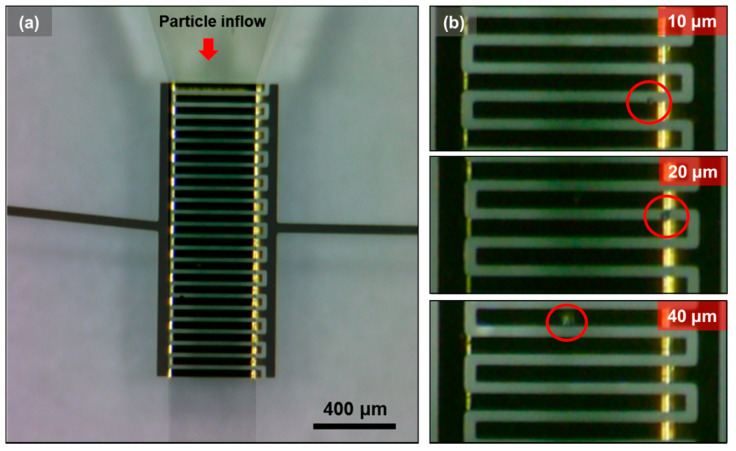
(**a**) Optical paragraph of the detection region marked with flow direction and (**b**) video frames of particles passing through by size (d = 10, 20, and 40 µm).

As shown in Figure 9a, particles passing through the IDEs with an optical microscope were recorded. Figure 9b shows particle samples of different sizes (10, 20, and 40 µm) passing through the detection region. Therefore, peak signals generated by the particles can be optically validated.

**Figure 10:**
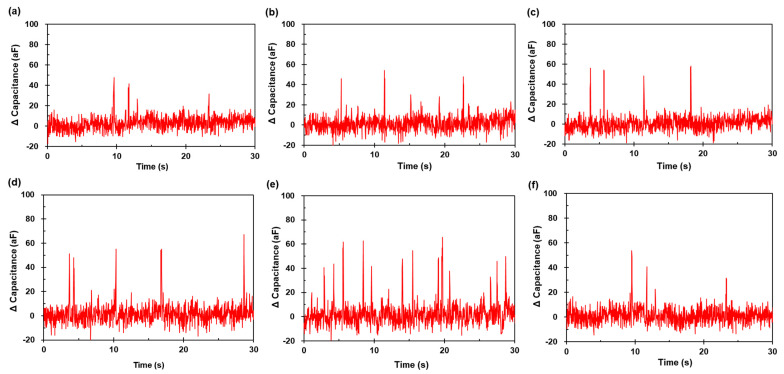
Measured capacitance signals for the aluminum particles (d=20 µm) under frequency of (**a**) 1.0 MHz, (**b**) 1.2 MHz, (**c**) 1.4 MHz, (**d**) 1.6 MHz, (**e**) 1.8 MHz, and (**f**) 2.0 MHz.

The test was conducted by sweeping the excitation frequency, because the dielectric properties depended on the applied AC frequency. Figure 10 presents the capacitance signal results for the 20 μm aluminum particles under different frequencies with 0.2 MHz intervals. The measured capacitance signals (ΔC) generated by each particle were identified using the recorded optical synchronization. The positive pulse immediately occurred as soon as the particle crossed the detection element. The data sampling rate was appropriate for tracking the particle trajectory. The amplitudes ranged from 31 aF to 47 aF (Figure 10a), 30 aF to 53 aF (Figure 10b), 43 aF to 57 aF (Figure 10c), 39 to 67 aF (Figure 10d), 38 aF to 66 aF (Figure 10e), and 31 aF to 53 aF (Figure 10f).

The root-mean-square noise ($N_{\mathrm{rms}}$) decreased from 6.53 aF to 5.63 aF depending on the frequency increment. To find the resonant frequency that increased the change in capacitance, the signal-to-noise ratio (SNR) was used, which can be expressed as

(14) $$\mathrm{SNR}=10\cdot \log_{10}(\frac{\bar{C}}{N_{\mathrm{rms}}})$$

where $\bar{C}$ is the average capacitance. Based on the 20 µm aluminum particle detection results, the SNR at 1.8 MHz was the highest at 9.89 compared to the other. Further, tests were performed at this frequency for particles of smaller (d=10 μm) and larger (d=40 μm) sizes to determine the amplitude tendency.

**Figure 11:**
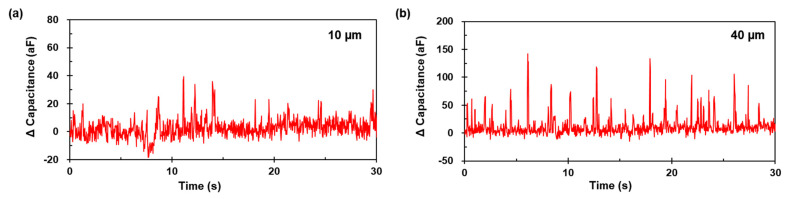
Measured capacitance signals for the aluminum particle sizes in (**a**) d=10 µm (**b**) d=40 µm at 1.8 MHz.

**Figure 12:**
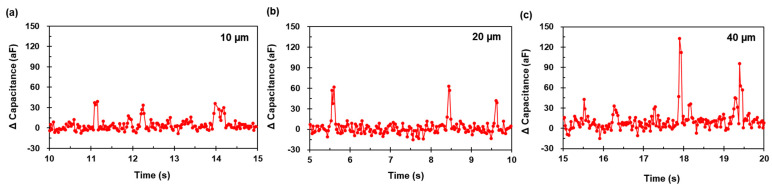
Close-up view of 5 s interval from measured capacitance signals for the aluminum particle sizes in (**a**) d=10 µm (**b**) d=20 µm (**c**) d=40 µm at 1.8 MHz.

Figure 11 presents the capacitance signal results for the 10 µm and 40 µm aluminum particles at 1.8 MHz. The amplitude ranges from 20 to 37 aF (Figure 11a) and 30 aF to 142 aF (Figure 11b), respectively. The results indicate that the particle detection element could detect individual particle sizes from 10 µm to 40 µm. As the data sampling point was at least four, even for different particle sizes, the operational conditions were adequately determined, as presented in Figure 12.

From the above results, the capacitance change tends to be proportional to the particles. This is because the volume occupied by the particle is directly related to the capacitance change. Of course, the range of the measured signal amplitude based on the particle size is wide. This is because of the influence of particle *z*-axis position from the IDEs, which have commonly been pointed out at the planar electrode. Additionally, the existence of particles smaller than provided added uncertainty in that one cannot know the exact particle size. Therefore, the relationship between the particle size and the capacitance change could be possible to estimate roughly, but accuracy is still limited.

The determined geometrical structure of the detection region and the element exhibited good synergies in terms of sensing sensitivity and particle delivery. The element can detect small-sized (d=10 µm) MPs using low-conductive media (oil) and the megahertz level of excitation frequency. Although the relationship between the particle size and the measured capacitance signal was not accurately derived due to particle *z*-axis position and inexact particle size, the tendency was identified. The detection element can be expected to achieve airborne MP detection using similar operating conditions of the test after being integrated. 

### 4.2. Performance of Integrated Microfluidic Airborne MP Detection Chip

#### 4.2.1. Microcirculation

Because our proposed system aims to perform automated and continuous airborne particle collection for detection, it must be ensured that the oil microcirculation is stable. If a mismatch occurs in the flow rate of microcirculation owing to a bottleneck, overflow occurs and causes the chip to fail. It is essential to monitor the circulation under an optical microscope. Because the validated particle detection element is also sensitive to measuring the fluid flux, the operating condition of the circulation can be identified in the measured capacitance.

The proposed system was connected to a pump system comprising a miniature vacuum pump (G6/01-K-LC, Thomas, Memmingen, Germany) and a peristaltic pump (9QX, Boxer, Ottobeuren, Germany). The microcirculation rate was the same as that used in the validation test (10 μL·min^−1^). The vacuum air pump used was 3 L·min^−1^. To visually confirm microcirculation, the region of the liquid surface was recorded.

**Figure 13:**
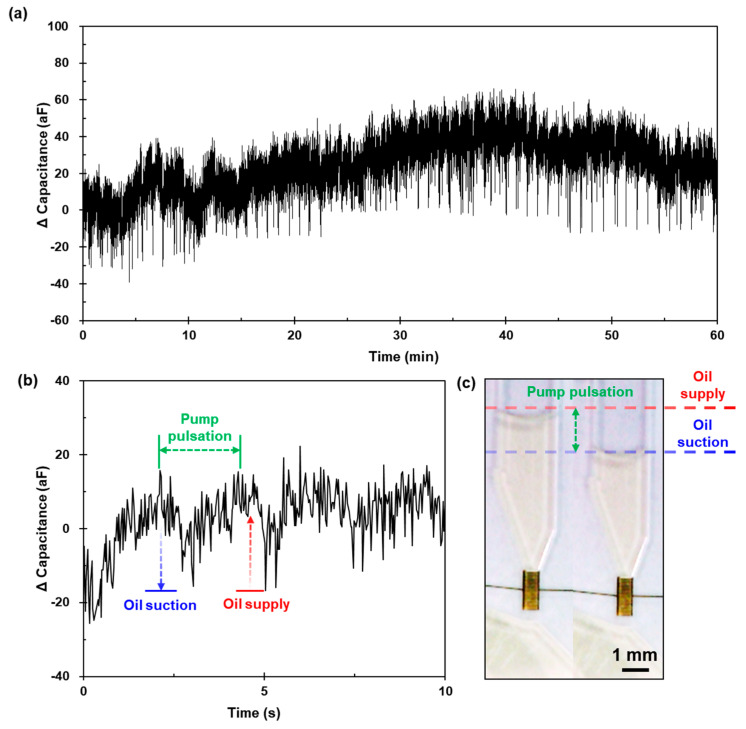
(**a**) Measured capacitance signals during operating the pump system for 60 min, (**b**) close-up view of 10 s interval, and (**c**) video frames showing the fluctuating liquid interface according to peristaltic pump operation.

Figure 13a presents the measured capacitance signals during the operation of the pump system for an hour. The periodic pattern in which the drifting signal descended and recovered was identified as ± 10 aF, as illustrated in Figure 13b. In addition, the surface level of the oil fluctuated, as illustrated in Figure 13c. This can be explained as a pump pulsation that occurred owing to the operation principle of the peristaltic pump. Squeeze (oil suction from the oil reservoir) for self-priming and rotation (oil supply to the oil inlet port) to force the oil onward by three rollers in the pump caused the internal pressure to increase and dissolve. Therefore, as the sensing volume was directly related to the signal, it expanded and contracted repeatedly owing to the PDMS elasticity.

These results demonstrate that the microcirculation in our microfluidic system operated stably. Although periodic patterns and signal drifting are confirmed, owing to pulsation, the impact is negligible for microcirculation in the microfluidic circuit. This is because the signal of the particles delivered by the microcirculation is discernible, compared to the typical amplitude of particle detection (ΔC≥30 aF). The proposed system maintains the liquid surface for collecting airborne particles based on the recorded image of the liquid surface.

#### 4.2.2. Real-Time and Continuous Monitoring of Airborne MPs on a Test-Bed Chamber

**Figure 14:**
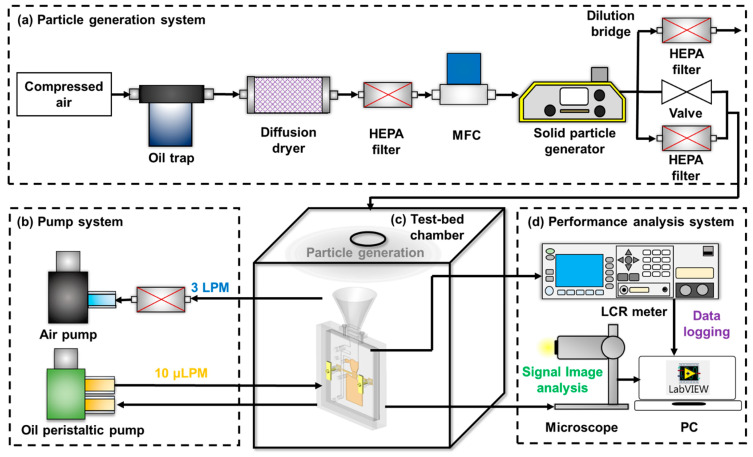
Schematic of the experimental set-up for performance evaluation of the proposed system; (**a**) particle generation system, (**b**) pump system, (**c**) test-bed chamber, and (**d**) performance analysis system.

The proposed system should be capable of responding to MPs generated in the air. Consequently, the performance of particle delivery from air to oil and real-time detection based on microcirculation must be continuous and sensitive. To evaluate the performance of the proposed system in an environment where particles are generated locally in the air, the experimental setup was established in four sections: (a) the particle generation system, (b) the pump system, (c) the test-bed chamber, and (d) the performance analysis system, as illustrated in Figure 14. Impurities (oil droplets, moisture, and unknown particles) in the compressed air were filtered through an oil trap, diffusion dryer, and high-efficiency particulate air (HEPA) filter. The mass flow controller (VIC-D200, MKP Co., Hwaseong, Korea) adjusted the flow rate of the purified air and supplied it to a solid particle generator (model SAG 410, TOPAS, Dresden, Germany). Airborne MPs (diameter of 40 µm) were then generated as the minimum conditions by the SAG. The instrument was connected to a dilution bridge to prevent particle overflow to the chip. The diluted particles transported by the air were injected into a 0.125 m^3^ test-bed chamber. In the test-bed environment, the device was connected to the pump system and a performance analysis system and then evaluated.

**Figure 15:**
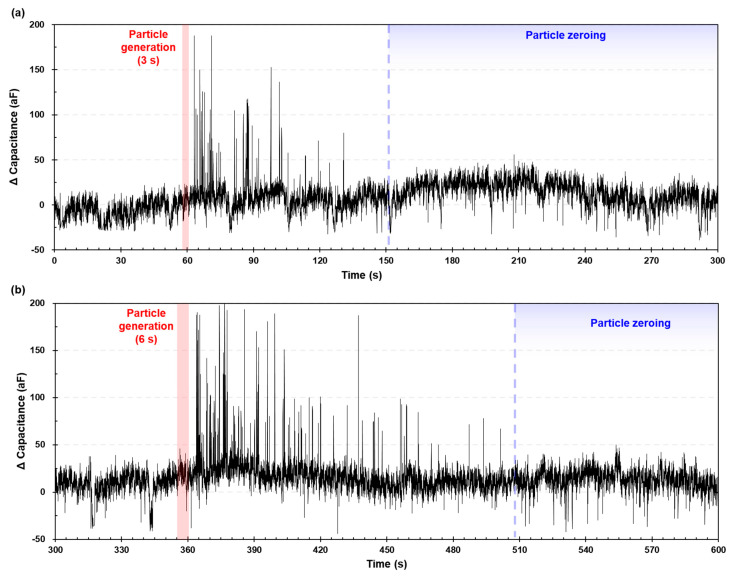
Performance evaluation of the proposed system in a test-bed condition; (**a**) 3 s and (**b**) 6 s.

Figure 15 illustrates the measured capacitance signal results for airborne MPs over 10 min. Particle generation was performed after no pulses were applied. At the beginning of the test, until 60 s without particle generation, there was no visible signal compared to the baseline level (~ 20 aF). Within a few seconds after particle generation, pulse signals rapidly occurred. The amplitudes ranged from 30 aF to 188 aF. After each particle generation event (3 s and 6 s, respectively), the proposed system could be restored to the particle zeroing conditions by purging the oil reservoir with microcirculation. Typical signals (≥ 130 aF) were detected, owing to the particle delivery mechanism, that is, particle collection and delivery in sequence. The reason could be considered to be particle *z*-axis position or coincidence error; multiple particles were counted as one (d≥40 µm) without being distinguished.

**Figure 16:**
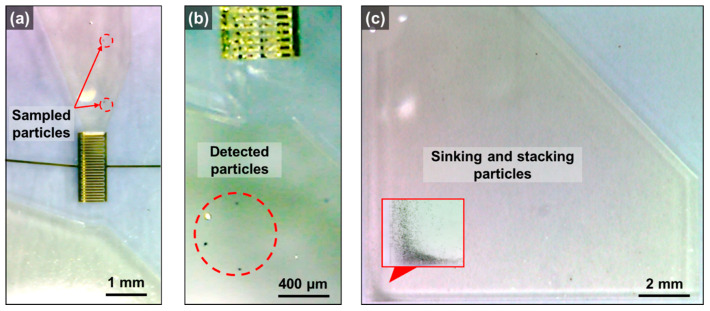
Video frames of particle delivery on the proposed system; (**a**) delivered particles at the inlet of the detection region, (**b**) detected particles at the outlet of the detection region, and (**c**) accumulated particles on the bottom of the oil reservoir.

The video frames show the microcirculation-based particle delivery performance, as presented in Figure 16. At the entrance of the detection region (Figure 16a), the particles that were sampled from the air to the liquid were delivered to the particle detection region. The measured particles escaped rapidly from the detection region and sunk to the bottom by losing their inertia (Figure 16b). Subsequently, the particles were seen to be sinking and stacking at the bottom of the oil reservoir (Figure 16c).

**Figure 17:**
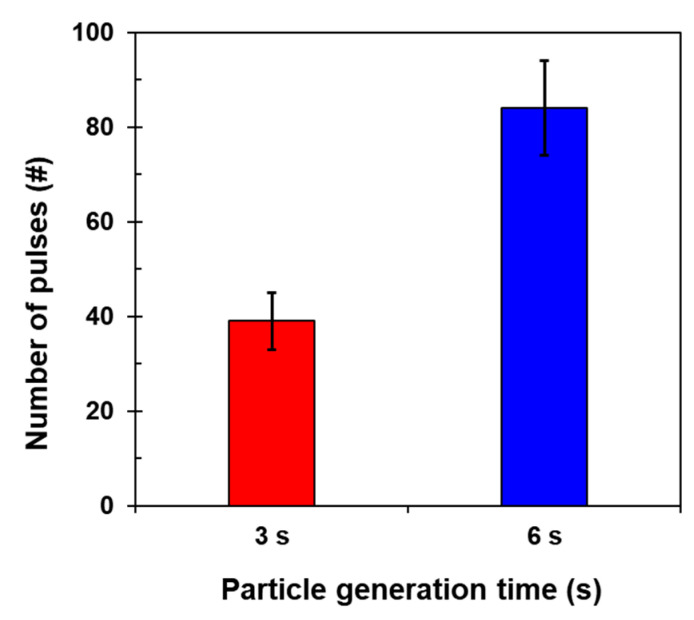
Number of capacitive pulses according to particle generation times (3 s and 6 s).

Figure 17 presents the number of capacitive pulses in different particle generation times. The pulses were counted based on the validated signal patterns of the individual particles. The amplitude criterion for distinguishing the noise level and pulse is ΔC ≥ 30 aF. Given the results of the validation test for the MPs in this study, this amplitude could be applied to understand the particle generation that suddenly occurred larger than a certain size. Reflecting the errors owing to particle behavior, the pulses were 39 and 84 for different particle generation times: 3 s and 6 s, respectively. The longer the particle generation time, the greater the number of pulse signals identified.

Consequently, the proposed system can perform particle delivery and detection with continuous microcirculation. The proposed system is applicable in an environment where the MPs are generated randomly, to detect micro-sized (from 10 µm to 40 µm) airborne MPs on a real-time basis. It can be used at any site where on-site airborne MP monitoring is required (e.g., semiconductor package chambers and battery manufacturing facilities).

## 5. Conclusions and Discussion

So far, many studies have been developed MEMS-compatible sensors to detect MPs. Specifically, sensors based on the inductive method have been recently developed to improve the sensitivity, in that they can distinguish ferromagnetic and non-ferromagnetic metal components [25,26,27]. However, they are not applicable for small-sized airborne MP detection due to their still low sensitivity to non-ferromagnetic metals. In addition, including developed capacitive sensors [28], both of them have been progressed and focused on the monitoring of oil contamination, not metal emission in the air. For our sensor, small-sized airborne MPs are monitored in real-time with continuous particle delivery and detection without periodical cleaning. A comparison between the current work with other MEMS-compatible MP detection sensors is summarized in Table 2.

A novel microfluidic airborne MP detection system was developed and demonstrated in this study. Owing to chip operation combining microcirculation-based particle-to-liquid collection with a capacitive sensing method onto the microfluidic platform, the proposed system could continuously monitor airborne MPs. The integrated microfluidic chip was realized on a single chip using a simple semiconductor fabrication process. Therefore, the proposed system is cost-efficient and field-portable; it is easy to use by simply exchanging the disposable chip after contamination.

Experiments were conducted to evaluate the performance of each function (particle detection and particle delivery) and the integrated system. The results indicated that the particle detection element can count the individual micro-sized MPs (at least 10 μm in diameter) in real-time with high sensitivity under an optimal excitation frequency (1.8 MHz). The proposed system integrated with the validated element exhibits signal fluctuation at the baseline, while the microcirculation was operated. However, this was insignificant compared to the level of MP detection. For each particle generation event in the test-bed chamber, the results indicated that the proposed system could detect airborne MPs in real-time and can be optically identified. Furthermore, the system can maintain continuous measurement conditions by purging itself for a while to respond to newly generated particle events. Therefore, the proposed system is capable of real-time and continuous monitoring. Herein, our sensor has limitations since it based on the microfluidic platform. Since the suction area is small and a high flow rate of vacuum air hampers the chip operation, it has the limitations on collecting a large number of airborne MPs. These limitations of this study are needed to be addressed in future work.

The possibility of on-site monitoring of the environment where airborne MPs are highly localized and unexpected can be realized with compactness and sensing performance. Thus, the proposed system can be further expected to be widely applicable in workplaces dealing with metal sources, because taking measures immediately and determining the causes of defect points is possible.

## Figures and Tables

**Table 1:** Design factors of the IDEs.

Design Factors	Value
Finger gap (g)	20 µm
Finger width (w)	40 µm
Finger length (l)	390 µm
Number of fingers (n)	13
Thickness (t)	0.21 µm
Total length ($T_{L})$	1480 µm
Total width ($T_{w})$	490 µm

**Table 2:** Comparison of current work with other MEMS-compatible MP detection sensors recently developed.

Detection Method	Detectable MP Range	Advantages	Disadvantages	Airborne MP Detection
Inductive [25]	60–240 μm copper	Distinguishes ferromagnetic and non-ferromagnetic metals	Low sensitivity to non-ferromagnetic metals	No
Inductive and Resistive [26]	46 μm iron 110 μm copper			No
Inductive [27]	20 μm iron 100 μm copper			No
Capacitive [28]	10–30 μm aluminum	High sensitivity	Low throughput	No
Capacitive(Current work)	10–40 μm aluminum	High sensitivity and self-cleaning	Low throughput	Yes
